# Supplementary material for: Phylogenetic Analysis of a Newcastle Disease Virus Strain Isolated from Domestic Poultry and Its Potential for Vaccine Development in the Republic of Kazakhstan
Source: Vaccines (Basel). 2025 Apr 23;13(5):440. doi: 10.3390/vaccines13050440 (PMC12116048; doi:10.3390/vaccines13050440)
Supplement: Supplementary file 1 [file vaccines-13-00440-s001.zip › supplementary material 1.pdf]

Supplementary Table S1: Clinical indicators in birds observed after challenge with a virulent NDV strain

Bird number	Group	Observation period, day									
		**1**	**2**	**3**	**4**	**5**	**6**	**7**	**8**	**9**	**10**
**1**	CG	0	1	1	2	3	4	0	0	0	0
**2**		0	1	2	3	4	0	0	0	0	0
**3**		0	1	1	3	4	0	0	0	0	0
**4**		0	1	2	3	4	0	0	0	0	0
**5**		0	1	1	3	4	0	0	0	0	0
**6**		0	1	2	3	4	0	0	0	0	0
**7**		0	0	1	3	4	0	0	0	0	0
**8**		0	1	2	2	3	4	0	0	0	0
**9**		0	1	2	3	4	0	0	0	0	0
**10**		0	0	1	2	3	4	0	0	0	0
**Average clinic score,point**		**0**	**0,8**	**1,5**	**2,7**	**3,7**	**1,2**	**0**	**0**	**0**	**0**
**11**	G3	0	0	0	0	0	0	0	0	0	0
**12**		0	0	0	0	0	0	0	0	0	0
**13**		0	0	0	0	0	0	0	0	0	0
**14**		0	0	0	0	0	0	0	0	0	0
**15**		0	0	0	0	0	0	0	0	0	0
**16**		0	0	0	0	0	0	0	0	0	0
**17**		0	0	0	0	0	0	0	0	0	0
**18**		0	0	0	0	0	0	0	0	0	0
**19**		0	0	0	0	0	0	0	0	0	0
**20**		0	0	0	0	0	0	0	0	0	0
**Average clinic score,point**		**0**	**0**	**0**	**0**	**0**	**0**	**0**	**0**	**0**	**0**
**21**	G4	0	0	0	0	0	0	0	0	0	0
**22**		0	0	0	0	0	0	0	0	0	0
**23**		0	0	0	0	0	0	0	0	0	0
**24**		0	0	0	0	0	0	0	0	0	0
**25**		0	0	0	0	0	0	0	0	0	0
**26**		0	0	0	0	0	0	0	0	0	0
**27**		0	0	0	0	0	0	0	0	0	0
**28**		0	0	0	0	0	0	0	0	0	0
**29**		0	0	0	0	0	0	0	0	0	0
**30**		0	0	0	0	0	0	0	0	0	0
**Average clinic score,point**		0	0	0	0	0	0	0	0	0	0

**Score**	**Clinical Symptoms**
0 points	No symptoms.
1 point	Loss of appetite or refusal to drink water.
2 points	Mild respiratory symptoms or greenish diarrhea.
3 points	Severe condition before death.
4 points	Fatal outcome.
