# Supplementary material for: Phylogenetic Analysis of a Newcastle Disease Virus Strain Isolated from Domestic Poultry and Its Potential for Vaccine Development in the Republic of Kazakhstan
Source: Vaccines (Basel). 2025 Apr 23;13(5):440. doi: 10.3390/vaccines13050440 (PMC12116048; doi:10.3390/vaccines13050440)
Supplement: Supplementary file 1 [file vaccines-13-00440-s001.zip › supplementary material 2.pdf]

Supplementary Table S2: Results of individual HI tests in birds after vaccination

№	Vaccine	№ bird	7 day	log 2	14 day	log 2	21 day	log 2	28 day	log 2
1	Monovalent Newcastle Disease Vaccine Based on PMV-1/Astana/chicken/49/98 Strain + Montanide ISA 70<br><br>Group 3	1	0	0	32	5	512	9	1024	10
		2	0	0	128	7	512	9	512	9
		3	0	0	64	6	512	9	1024	10
		4	0	0	64	6	512	9	512	9
		5	0	0	8	3	512	9	512	9
		6	0	0	16	4	1024	10	1024	10
		7	0	0	64	6	512	9	512	9
		8	0	0	32	5	512	9	512	9
		9	0	0	32	5	512	9	256	8
		10	0	0	64	6	1024	10	1024	10
GMT			0	0	37,3289293	5,16868	552,990586	9,19166	597,262868	9,27748
SE			0	0		0,40		0,1		0,2
2	Monovalent Newcastle Disease Vaccine Based on PMV-1/Astana/chicken/49/98 Strain + Montanide ISA 78<br><br>Group 4	11	0	0	512	9	1024	10	1024	10
		12	0	0	512	9	1024	10	1024	10
		13	0	0	512	9	1024	10	1024	10
		14	0	0	512	9	1024	10	2048	11
		15	0	0	256	8	512	9	512	9
		16	0	0	1024	9	512	9	512	9
		17	0	0	1024	9	512	9	512	9
		18	0	0	1024	9	1024	9	1024	10
		19	0	0	512	9	512	9	512	9
		20	0	0	512	9	1024	10	1024	10
GMT			0	0	597,2628683	8,89462	752,50406	9,48683	812,7493386	9,67913
SE			0	0		0,1		0,1		0,2
